# Finite Element Analysis of Pelvic Floor Biomechanical Models to Elucidate the Mechanism for Improving Urination and Defecation Dysfunction in Older Adults: Protocol for a Model Development and Validation Study

**DOI:** 10.2196/56333

**Published:** 2024-05-31

**Authors:** Rui Wang, Guangtian Liu, Liwei Jing, Jing Zhang, Chenyang Li, Lichao Gong

**Affiliations:** 1 School of Nursing Capital Medical University Beijing China; 2 College of Nursing and Rehabilitation North China University of Science and Technology Hebei China; 3 Neurology Department of Xuanwu Hospital Capital Medical University Beijing China

**Keywords:** elderly, older adults, pelvic cavity, finite element analysis, biomechanical model, protocol, urination, incontinence, aging, bowel dysfunction

## Abstract

**Background:**

The population is constantly aging, and most older adults will experience many potential physiological changes as they age, leading to functional decline. Urinary and bowel dysfunction is the most common obstacle in older people. At present, the analysis of pelvic floor histological changes related to aging has not been fully elucidated, and the mechanism of improving intestinal control ability in older people is still unclear.

**Objective:**

The purpose of this study is to describe how the finite element method will be used to understand the mechanical characteristics of and physiological changes in the pelvic cavity during the rehabilitation process, providing theoretical support for the mechanism for improving urination and defecation dysfunction in older individuals.

**Methods:**

We will collect magnetic resonance imaging (MRI) and computed tomography (CT) data of the pelvic cavity of one male and one female volunteer older than 60 years and use the finite element method to construct a 3D computer simulation model of the pelvic cavity. By simulating different physiological states, such as the Valsalva maneuver and bowel movement, we will verify the accuracy of the constructed model, investigate the effects of different neuromuscular functional changes, and quantify the impact proportions of the pelvic floor muscle group, core muscle group, and sacral nerve.

**Results:**

At present, we have registered the study in the Chinese Clinical Trial Registry and collected MRI and CT data for an older male and an older female patient. Next, the construction and analysis of the finite element model will be accomplished according to the study plan. We expect to complete the construction and analysis of the finite element model by July 2024 and publish the research results by October 2025.

**Conclusions:**

Our study will build finite element models of the pelvic floor of older men and older women, and we shall elucidate the relationship between the muscles of the pelvic floor, back, abdomen, and hips and the ability of older adults to control bowel movements. The results of this study will provide theoretical support for elucidating the mechanism for improving urination and defecation dysfunction through rehabilitation.

**Trial Registration:**

Chinese Clinical Trial Registry ChiCTR2400080749; https://www.chictr.org.cn/showproj.html?proj=193428

**International Registered Report Identifier (IRRID):**

DERR1-10.2196/56333

## Introduction

With the aging population, approximately 1.4 billion people were aged 60 years and older in 2022, which is expected to double by 2050 worldwide [1]. The majority of older people experience numerous underlying physiological changes with increasing age, leading to functional decline. Urination and defecation dysfunction are the most common impairments in older age. In China, the prevalence of urinary incontinence was 74.8% among older women [2] and 54.4%-60.0% among hospitalized older patients [3,4], while older men are at a higher risk of severe fecal incontinence than older women [5]. Furthermore, a systematic review and meta-analysis reported that the global prevalence of constipation in older adults was 18.9% (95% CI 14.7%-23.9%) [6]. Urination and defecation dysfunction play a crucial role in determining care dependence among older adults. Further, these factors contribute to the increase in susceptibility to negative emotions in older adults and burden among their caregivers [7]. Therefore, addressing the toileting needs of older adults is a pressing global challenge that requires immediate attention and a comprehensive solution.

Factors affecting the ability to control urination and defecation are mainly divided into 3 parts, including the pelvic floor muscle group, core muscle group, and sacral nerve. Rehabilitation training methods, including pelvic floor muscle training, biofeedback, electrical stimulation, magnetic stimulation, and vibrational stimulation, can enhance neural regulatory ability and muscle strength, thus improving dysfunction in urination and defecation [8,9]. The rehabilitation training methods and intensity are quantitatively related to neural control and muscle strength parameters. It is crucial to quantify this relationship to explain the mechanism underlying improvement in dysfunction in urination and defecation. At present, an analysis of histological changes in pelvic floor tissue related to aging has not been fully elucidated; hence, the mechanism underlying enhancement of bowel control in older adults remains unknown.

Finite element analysis is a technique to simulate the mechanical properties of an object by dividing it into discrete elements and creating a numerical calculation model to represent its behavior [10], and it could potentially reveal the mechanism for improving bowel control. Therefore, we aim to use a finite element model to identify the relationship among rehabilitation training methods, intensity, and parameters related to neural control and muscle strength, ultimately determining the mechanism by which urination and defecation dysfunction can be improved in older people.

## Methods

### Ethical Considerations

This study has been approved by the Biomedical Ethics Committee of Capital Medical University on June 5, 2023 (registration number 2023-079). The implementation of this study complied with relevant ethical requirements, and the study participants signed informed consent forms before undergoing medical imaging. Participation in this study is completely voluntary, and the participants may refuse to continue participating in this study at any time without any negative impact on their medical care. We shall retain all information about the study participants, including their identity, general information, medical history, results of magnetic resonance imaging (MRI) and computed tomography (CT) imaging, etc. Only authorized researchers, ethics committees, and research project approval departments can access records related to this study. The names of research participants will not appear in any public materials, reports, or articles related to this research. The study participants receive free pelvic floor MRI and CT examinations in this study, and upon completion of data collection, they will receive a corresponding compensation of 1000 CNY (approximately US $138).

### Patient Involvement

**Figure 1:**
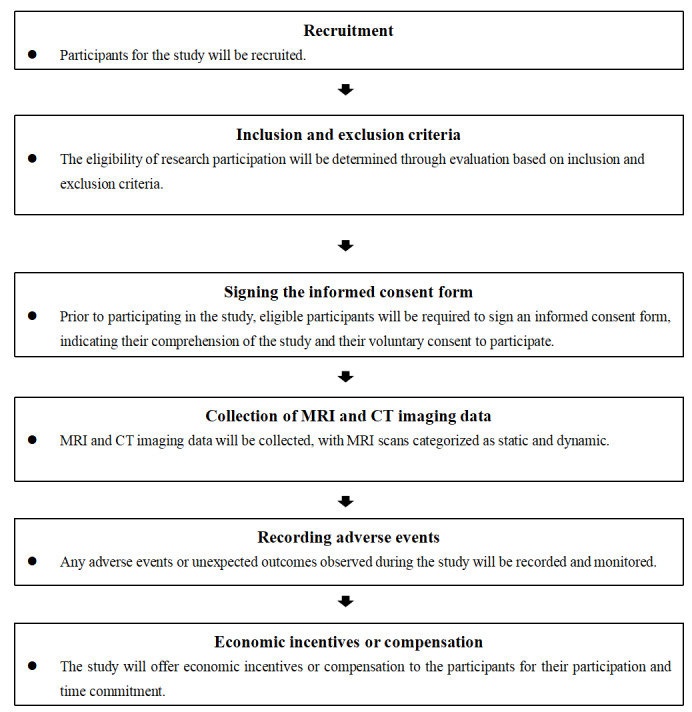
Flowchart of the participant recruitment process. CT: computed tomography; MRI: magnetic resonance imaging.

In this study, one man and one woman both aged ≥60 years will be included. Eligible participants are required to have good physical health, normal daily living abilities, normal cognitive abilities, normal communication skills, normal pelvic floor function, and no urination and defecation dysfunction (Figure 1). Inclusion and exclusion criteria are outlined in Multimedia Appendix 1.

Clinical data will be collected at a hospital in Beijing. Demographic information will be collected, encompassing gender, age, BMI, and the frequency and characteristics of daily bowel and bladder movements. Their health conditions will be also captured, consisting of hypertension, diabetes, heart disease, stroke, Alzheimer disease, arthritis, osteoporosis, chronic obstructive pulmonary disease, tumors, and vision or hearing problems.

### Software and Hardware

The computer CPU has an Intel (R) Core (TM) i5 processor, with a 64-bit operating system, 16 gigabytes (GB) of memory, 500-GB hard disk, and 900-GB external hard disk. The software involved in medical imaging analysis include Mimics (Materialise NV), Geomagic Studio (3D Systems Inc), and Abaqus (Dassault Systèmes). Mimics and Geomagic Studio are used for 3D model building; Abaqus is used for 3D model processing and biomechanical finite element analysis.

### Collection of MRI and CT Image Data

Participants will be scheduled for MRI and CT imaging, with the goal of acquiring medical imaging data pertaining to the organs of the pelvis, bones, and muscles associated with defecation and urination.

Due to the similar density of pelvic muscles, fascia, and other tissues, distinguishing them in CT and ultrasound imaging is challenging. MRI is the most recognized and important imaging modality [11]. However, CT has a higher resolution for bone tissue, so CT scanning of pelvic bones and MRI scanning of pelvic floor muscle tissue are used for medical imaging. The data from both scans are combined to construct a finite element model of the study participants’ pelvic cavity. Multimedia Appendix 2 provides detailed descriptions of the steps and receipt collection methods for static MRI, dynamic MRI, and CT imaging. Dynamic MRI will include Kegel movements and Valsalva maneuvers, providing a comparison for subsequent finite element model validation. Images will be acquired by skilled doctors from the MRI and CT departments, and the collected imaging data will be evaluated by radiologists with ≥5 years of experience in diagnosis using pelvic MRI and CT imaging. In cases of poor image quality, a rescan will be performed to obtain a clear image.

### Establishment of 3D Images

#### Data Import

**Figure 2:**
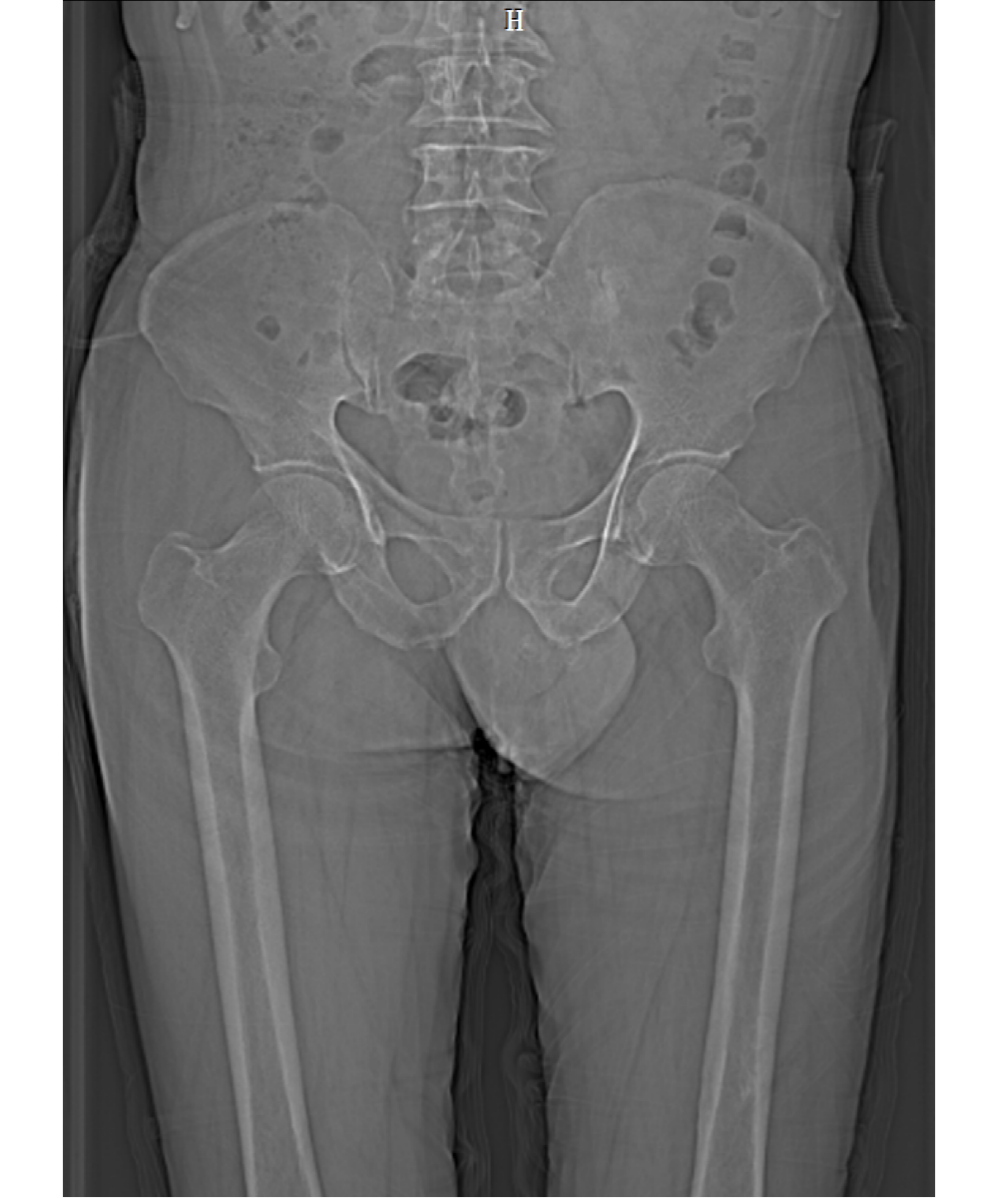
Magnetic resonance image of the older male participant. H: head.

**Figure 3:**
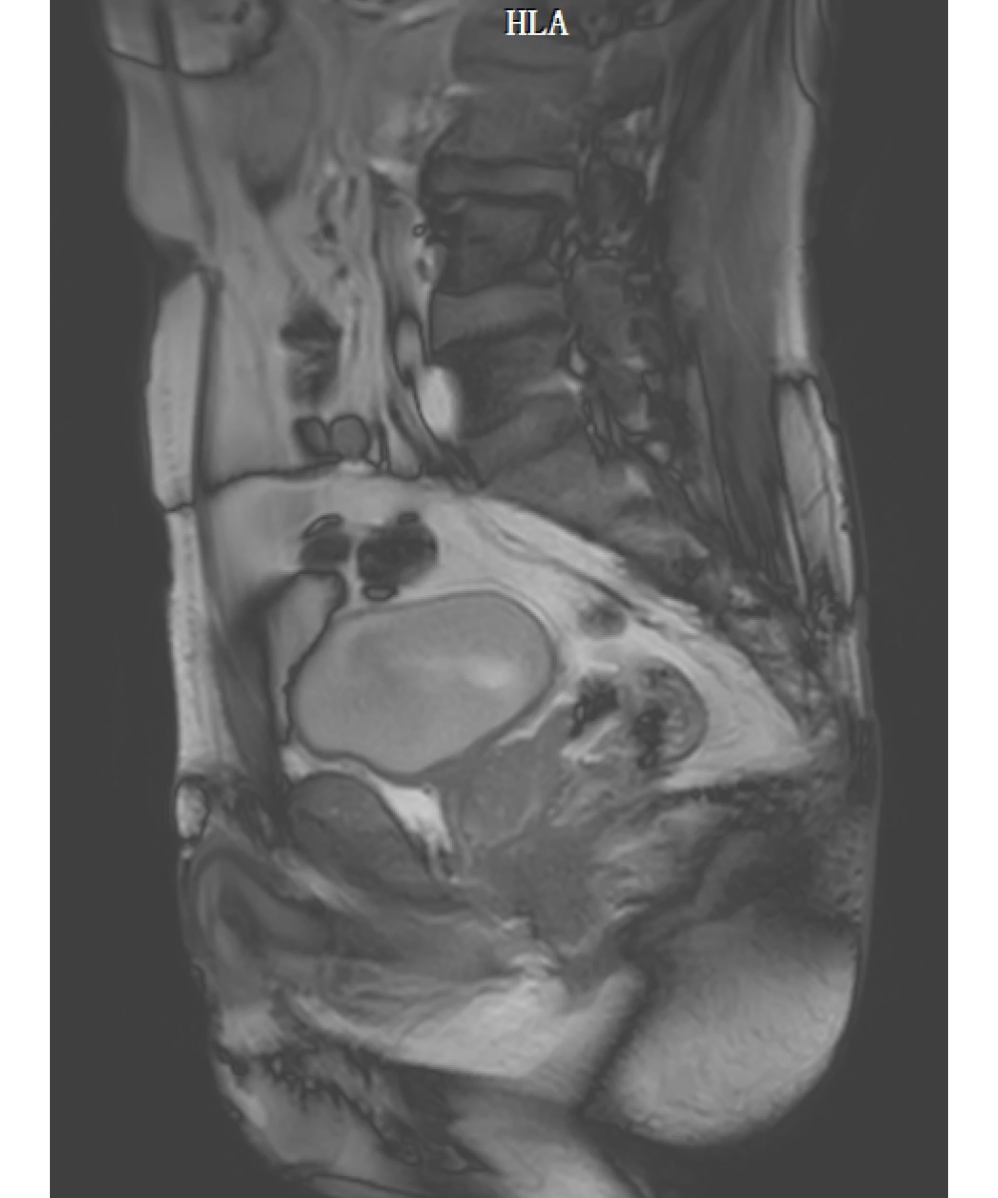
Computed tomography image of the older male participant. HLA: head left anterior.

**Figure 4:**
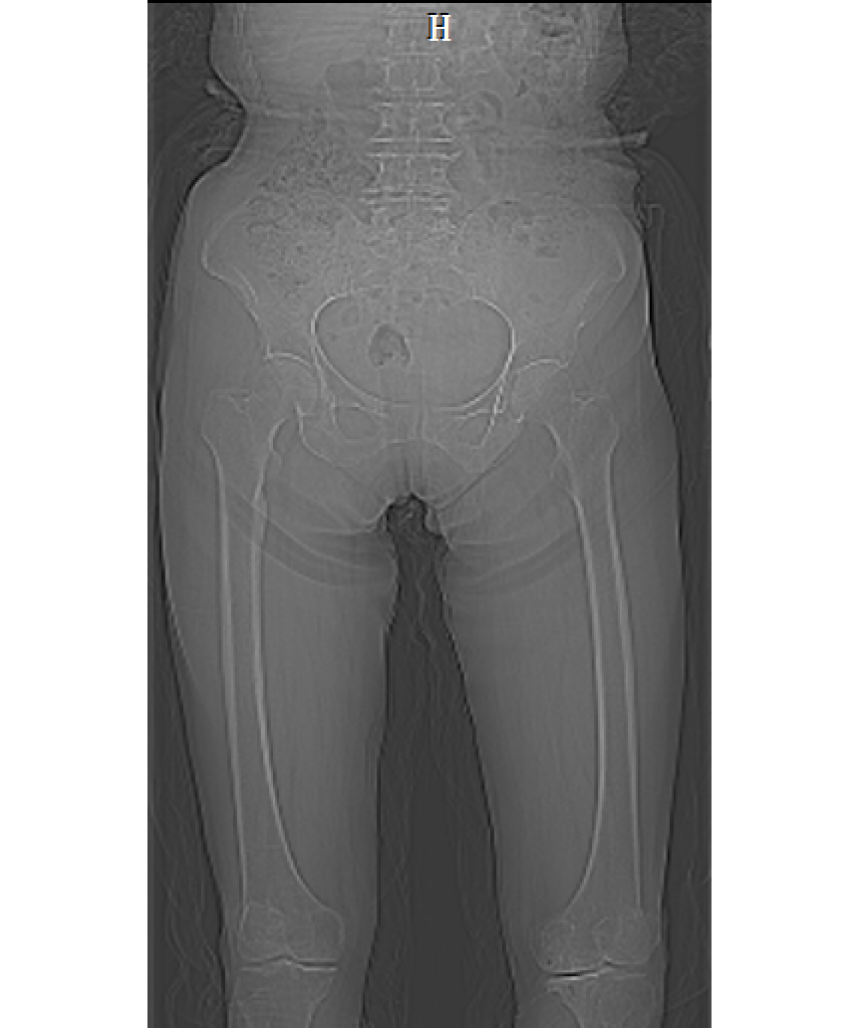
Magnetic resonance image of the older female participant. H: head.

**Figure 5:**
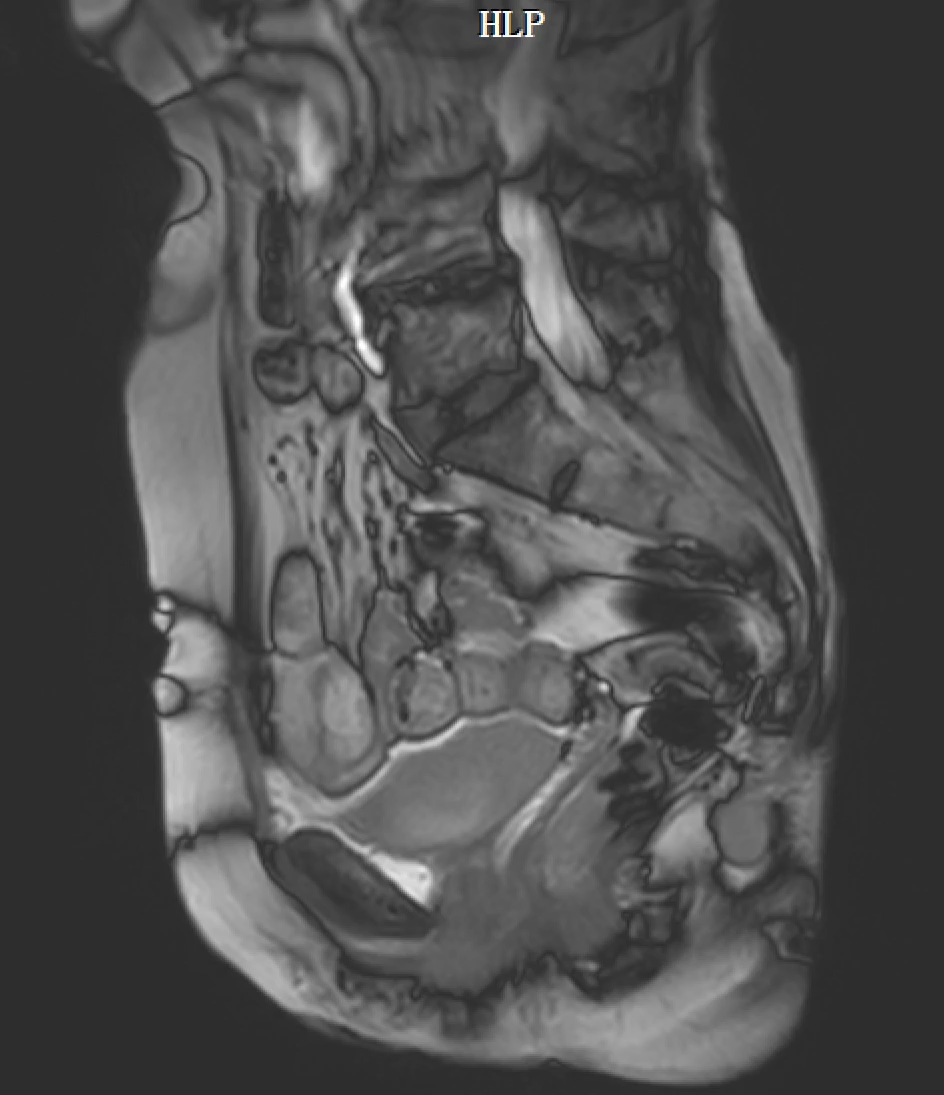
Computed tomography image of the older female participant. HLP: head left posterior.

The original MRI and CT imaging data will be imported into the medical image analysis software Mimics. This software will automatically generate the 3 views: cross-sectional, coronal plane, and sagittal plane views. To ensure optimal 3D reconstruction results, we will select the sagittal plane corresponding to the MRI and CT scanning section for further editing. CT and MR images of the older man and woman are shown in Figures 2-5.

#### Selecting Thresholds and Segmenting Images

The brightness and contrast of 2D images will be adjusted. Additionally, 2 experienced radiologists specializing in diagnosis using pelvic MRI and CT imaging will collaboratively outline the organs, bones, muscles, and muscle groups in each cross-sectional image to reach a consensus. When accessing the designated interface, we will perform threshold adjustments to accurately select the required organs, bones, muscles, and muscle groups. A self-set threshold method will be used to maximize the selection of these structures while minimizing interference from adjacent tissues. We will first use CT image data to outline skeletal parts including the hip bone, sacrum, coccyx, and femur, and then use MRI data to outline the pelvic floor muscle group, abdominal muscle group, back muscle group, hip muscle group and organ parts. The final data will combine bone parts, muscle group parts, and organ parts.

#### Generation of 3D Images

We will use the calculate 3D function in Mimics to directly obtain the corresponding 3D solid geometry model including organs, bones, muscles, and muscle groups, and then the model will be exported in stereolithography format.

#### Edge Processing

The initially created 3D image is relatively rough and will be enhanced through the smoothing function in Mimics to reduce the angles of points or edges, making the edges smoother, improving the overall appearance of the 3D image.

### Establishment of the 3D Model

We will import the 3D image into the reverse engineering software Geomagic Studio and then generate a solid 3D model of the pelvic cavity, which can be used for finite element analysis through computer gridding and surface fitting. The final 3D pelvic floor model will include several parts such as the pelvis, bladder, urethra, uterus, vagina, rectum, levator ani muscle, perineum, and uterosacral ligaments.

### Validation of Finite Element Models

During the finite element modeling process, it will be essential to validate the effectiveness of the finite element model. We will adopt 2 methods to validate the finite element model. The first method is the geometric verification method, we will compare the dynamic effects simulated by the established finite element model of the pelvic floor with the pelvic floor movements performed by actual subjects during dynamic MRI, for example, Kegel movements and Valsalva maneuvers, to determine whether the internal pressure and bladder deformation align. The second method is the validation of results obtained from the literature. The model will be validated through comparison with previous literature.

### Finite Element Model Mechanism Analysis

#### Building the Finite Element Model

##### Import a 3D Model

We will import the Standard ACIS Text file into Abaqus and convert it to a mesh model.

##### Volume Meshing

Volume meshing is crucial in constructing finite element models and conducting numerical simulations. Due to the complexity of the solid model that needs to be meshed, partitioning it into regular volume meshes is challenging. Therefore, we will use Abaqus’ free meshing method, which automatically generates a hybrid mesh consisting of triangles, tetrahedra, and hexahedra by dividing different tissues. The mesh will be divided into different forms based on the anatomical characteristics of each pelvic floor structure. If a satisfactory grid cannot be constructed for the local area, the model is locally fine-adjusted and iterated repeatedly until a high-quality grid is obtained.

##### Assignment of Material Properties

Once the 3D solid model is constructed, Abaqus will be used to assign mechanical properties, such as the elastic modulus, the Poisson ratio, element type, number of elements, and ligament strength to each constituent element, resulting in a model with defined mechanical characteristics. This study aims to determine the material characteristic parameters of the pelvic organ, skeleton, individual muscle and muscle groups, as well as each ligament, using relevant literature sources.

#### Constraining of Boundaries

When analyzing static structural mechanics, the muscles are in close proximity to various tissues. To replicate the model’s deformation under specific loads, the boundary must be adequately constrained to prevent nonconvergence caused by displacement uncertainty. The z-axis displacement of all nodes in the finite element model of the pelvic floor system should be restricted, and base constraints should be applied to the vagina, perineal body, rectum, levator ani muscle, pubic bone, and sacrococcygeal bone.

#### Application of Loads

Solid 3D elements can be used in static structural mechanics analysis. The surface loads from the participants can be applied to the finite element model’s surface. The model can undergo finite element analysis under different states and various load patterns. Integrating neuromuscular physiological information and medical imaging data into computer simulation models allows for simulating the effects of various stimulation methods, locations, and intensities on urinary and bowel control.

Currently, there are 5 known rehabilitation training methods for improving bowel function including pelvic floor muscle training, biofeedback, electrical stimulation, magnetic stimulation, and vibration stimulation. We have summarized the muscles corresponding to 5 types of rehabilitation training based on the literature contained in Multimedia Appendix 3. After completing the construction of the finite element model, we will conduct specific finite element analysis under different simulation plans. We will simulate the effects of muscle enhancement or weakening corresponding to the 5 types of rehabilitation training. The study simulated a 25%, 50%, 75%, 100%, normal, 25%, 50%, 75%, 75%, and 95% increase in muscle capacity by changing the correlation coefficient of elastic modulus to 1.25 times, 1.5 times, 1.75 times, 2 times, 1 time, 0.75 times, 0.5 times, 0.25 times, and 0.05 times, respectively. The main outcome measure of the study is the Mises stress cloud map and stress region of the area of interest in the finite element model of older adults under different simulation plans. The second outcome comprises the retrovesical and anorectal angles used to reflect the ability of older people to control their bowel movements. Among them, the retrovesical angle is the angle formed by the median sagittal plane of the bladder floor and the long axis of the urethra, and the anorectal angle is the angle between the longitudinal axis of the anal canal and the posterior wall of the rectum above the levator ani muscle.

**Figure 6:**
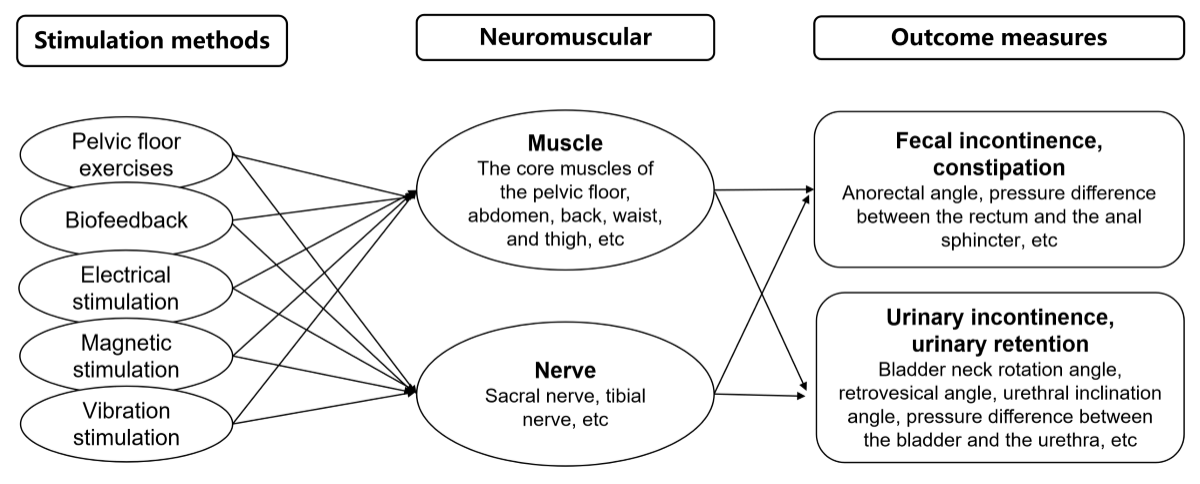
Schematic diagram of the mechanisms of enhanced defecation ability.

By comparing the changes in outcome measures under different parameter settings, our objective is to maximize the improvement of urination and defecation dysfunction while identifying the optimal muscle and neural parameters. This analysis will help quantify the relationship between rehabilitation training coordination, neural control, muscle strength, and other relevant parameters (Figure 6). It will provide insights into the mechanisms underlying the improvement of urination and defecation dysfunction in older adults.

## Results

We have registered the study in the Chinese Clinical Trial Registry (ChiCTR2400080749). After obtaining ethical approval, we recruited the study participants. As of January 13, 2024, we have completed the collection of MRI and CT imaging data for an older man and woman. Thereafter, the finite element model will be established as intended. We expect to complete the construction and analysis of the finite element model by July 2024 and publish the research results by October 2025.

## Discussion

### Principal Findings

Our study aims to generate finite element models of the pelvic floor of older men and women and derive the relationship among the muscles of the pelvic floor, back, abdomen, and hips and the ability of older adults to control their bowel movements. Pelvic floor issues significantly impact the quality of life and overall health of older adults, which plays a crucial role in determining care dependence among them. Given the most common problem of urination and defecation dysfunction among older people, which has still not been effectively addressed from a mechanistic perspective, our study will innovatively adopt finite element analysis to establish, validate, and further analyze the finite element model for pelvic floor function to address the aforementioned issues.

Finite element analysis is a reliable research method that has been verified by many studies [12-16], and it can reflect the condition of the human body. Finite element analysis has been widely adopted for urethral support function [17], injury in levator ani muscles, occurrence of pelvic floor diseases during vaginal delivery [18], and assessing the mechanism of posterior vaginal prolapse [19]. The finite element model can simulate biomechanical processes, avoiding the implementation of a complex interventional study. For our study, finite element models of urinary incontinence, urinary retention, constipation, and fecal incontinence for older adults will be established to simulate their real pelvic floor tissue and muscles. When designing and developing the finite element model, we shall consider simulating the structure and function of the real human body as much as possible. Professional doctors experienced in imaging diagnostics will outline the bones and muscles based on actual high-resolution MRI and CT imaging data to ensure consistency between the constructed finite element model and real human body structure as much as possible. In addition, we will also invite relevant experts in anatomy, rehabilitation medicine, physiology, bioengineering, and nursing to guide our subsequent finite element analysis to ensure that we can truly reflect the structure, function, and existing problems of the pelvic floor of older adults. It is notable that some studies on finite element models of the pelvic floor for urinary incontinence only recruited young postpartum women [20,21], which might have had an impact on the models’ adaptability. Older people are at a higher risk of incontinence and constipation due to decreased muscle strength, relaxation and reduced elasticity of pelvic floor tissues, and hormonal level alterations than their younger counterparts. Consequently, variations may arise in the anatomical structure, muscle tone, and elasticity of the pelvic floor, as well as in the material properties of the finite element model. There is currently a scarcity of pelvic floor finite element models for analyzing fecal incontinence, constipation, urinary incontinence, and urinary retention in older men and women, and our study can bridge this gap.

In addition to model construction, this study also intends to validate the effectiveness of the constructed model, which has been overlooked in many studies [22]. The validation part is crucial as it is closely related to the accuracy of the results of subsequent finite element analysis. Finite element analysis allows for quantitative assessment of the adaptability of pelvic floor structures to various mechanical loads. To address research questions, the finite element model can simulate 5 different rehabilitation training methods, including exercise training, biofeedback, magnetic stimulation, electrical stimulation, and vibration stimulation. By comparing the changes in the outcome indicators of defecation under different parameter settings, the optimal muscle and nerve parameters are determined to maximize the improvement of defecation dysfunction. This quantifies the relationship between the collaborative rehabilitation training methods and parameters such as neural control and muscle strength and reveals the mechanism underlying enhancement of control of defecation in older adults [11]. A realistic pelvic model based on MRI of asymptomatic women was established by Peng et al [17], wherein the combined weakening effect of the 3 components of the levator ani muscle exceeded the sum of the weakening effects caused by each individual component. The levator ani muscle group demonstrated good coordination by providing urethral support in a nonlinear additive manner. Therefore, it is reasonable to speculate that the interaction between muscles and nerves involves a more complex correlation rather than just a linear additive relationship. Our research will explore how to maximize the enhancement of pelvic floor muscle and nerve function in older adults to further improve urinary and bowel dysfunction. Research findings in the finite element analysis section will offer novel evidence to comprehend the physiological changes and mechanical characteristics of the pelvic cavity in older individuals, as well as provide theoretical support for elucidating the mechanisms underlying the improvement in urination and defecation dysfunction among them. Future studies can explore the potential of using the computer simulation model of the pelvic floor, as detailed in this paper, in the advancement of novel technologies for diagnosing and treating pelvic diseases in older individuals. In addition, our results can be extended to 3D printing and virtual reality systems to assist teaching practice. These would serve as key steps for transforming our research findings into practical applications.

This study describes a logical, rigorous, and scientific research protocol. However, the limitation of this study is the absence of statistical analysis. Due to individual variations, data on pelvic floor anatomy from the 2 participants may not comprehensively reflect the changes and characteristics observed in the broader population of older adults. Nevertheless, in initial stages of the study or due to resource constraints, the MRI and CT data from these 2 participants can be used to generate a preliminary pelvic model. Future studies can design and construct finite element models that contain more pelvic floor data of older adults. Alternatively, selecting different populations of older adults, such as those with urinary incontinence or those with disabilities, may be the next research direction.

### Conclusions

Finite element analysis offers the advantages of low risk, cost-effectiveness, and quantifiable metrics. This study encompasses the construction and validation of finite element models, as well as the analysis of their mechanisms. Our results will elucidate the correlation between muscular function and bowel control ability. Our findings will contribute theoretical insights into the mechanisms underlying enhancements in urinary and fecal control through rehabilitation, ultimately aiming to improve the quality of life of older adults.

## Appendix

Multimedia Appendix 1 Study eligibility criteria.

Multimedia Appendix 2 Scanning methods of static magnetic resonance imaging (MRI), dynamic MRI, and computed tomography.

Multimedia Appendix 3 Muscles corresponding to five rehabilitation trainings for four types of urinary and defecation dysfunction.

## Abbreviations

CT: computed tomography
GB: gigabyte
MRI: magnetic resonance imaging

## Data Availability

The data sets generated and analyzed during this study will be made available by the corresponding author on reasonable request.
