# Supplementary material for: Finite Element Analysis of Pelvic Floor Biomechanical Models to Elucidate the Mechanism for Improving Urination and Defecation Dysfunction in Older Adults: Protocol for a Model Development and Validation Study
Source: JMIR Res Protoc. 2024 May 31;13:e56333. doi: 10.2196/56333 (PMC11179018; doi:10.2196/56333)
Supplement: Multimedia Appendix 1 [file resprot_v13i1e56333_app1.docx]

**Table 1 Study eligibility criteria**

| **Inclusion criteria** | |
| --- | --- |
| Age qualification | ≥60 years old; |
| Healthy | Excluding diseases such as malignant tumors, diseases requiring hemodialysis, lymphoma, and other conditions that impact survival time; |
| The ability of daily living is normal | Subjects’ daily living ability will be evaluated using the Barthel Index, and only subjects with a score of 100 will be included in the study.. The Barthel Index, developed by Florence Mahoney and Dorothy Barthel, consists of 10 items and has a maximum score of 100. The Barthel Index demonstrates a Cronbach α coefficient >0.92, indicating its reliability. It is commonly employed to assess the daily living ability of patients with stroke, fractures, and geriatric conditions, and it finds widespread use in the field of rehabilitation medicine [23]. A Barthel index score of ≤40 indicates severe dependence, with subjects needing full assistance from others.; A total score of 41-60 indicates moderate dependence, with most subjects necessitating assistance from others.; A total score of 61-99 indicates mild dependence, with a small portion requiring care from others; A total score of 100 points indicates independence, and subjects do not require care from others; |
| The ability of cognitive is normal | This study will utilize the Beijing version of the Montreal Cognitive Assessment Scale (MoCA-BJ) to evaluate, subjects with a score greater than 20 in illiterate education level,, subjects with a score greater than 24 who have an elementary school education level, subjects with a score greater than 27 who have a middle school education level will be included. MoCA-BJ assesses visual-spatial function, naming, attention, repetitive sentences, fluency, abstract ability, delayed recall, and directional ability, with a maximum score of 30 points. When subjects with a score of 18-20 in illiterate education, subjects with a score of 21-24 in primary school education, subjects with a score of 25-27 in middle school education or higher, it indicates a decline in cognitive function. At the recommended cutoff value of 26 points, the sensitivity and specificity of this scale are 90.4% and 31.3% [24], indicating good reliability and validity; |
| Normal bowel movements and urination | The daily excretion patterns of stool and urine, including frequency and appearance, are normal; |
| The pelvic floor function is normal | This study will assess the normalcy of pelvic floor function using questionnaires.. For older women, we plan to use the Pelvic Floor Distress Inventory Short Form 20 (PFDI-20) to assess related symptoms and their impact on the participants' pelvic floor, bowel, and bladder. It is widely used internationally and suitable for middle-aged and elderly female patients in both clinical outpatient and inpatient settings.. Since 2007, scholars have applied it to women in late pregnancy and postpartum stages to explore the risk factors, incidence, and severity of pelvic floor dysfunction in postpartum women, its impact on quality of life, and the evaluation of the effectiveness of pelvic floor muscle exercise. The PFDI-20 consists of 20 items and 3 subscales, including the Urogenital and Reproductive Distress Inventory (UDI, 6 items), Pelvic Organ Prolapse Inventory (POPDI, 6 items), and Colorectal Anorectal Distress Inventory (CRADI, 8 items). Each item is rated on a 5-point scale, ranging from 0 to 4 points. A score of 0 indicates no symptoms (asymptomatic), while a score of 1 suggests symptomatic but no interference with daily life. Scores of 2-4 reflect varying degrees of symptoms and their impact on daily life. The average score of the subscale (the sum of scores for each item in the subscale/number of items) × 25 represents a score of 0-100 points on this subscale, and the total score of 0-300 points is obtained by adding the scores of the three subscales. The higher the score, the higher the degree of distress of PFD symptoms in patients [25]. This study included participants with a score of fewer than 75 points, and the severity level of each item in the final questionnaire should not exceed the level of "symptomatic but not life-threatening". Cronbach's of the total quantity table α The coefficient is 0.879, and the subscale Cronbach's α The coefficient ranges from 0.790 to 0.862, indicating a high degree of homogeneity and intrinsic correlation among the items in the scale. The retest reliability of the total scale is 0.776, indicating good stability of the scale. The sensitivity analysis results indicate that the questionnaire has good sensitivity;  For older males, we plan to use the International Consultation on Incontinence Modular Questionnaire for Male Lower Urinary Tract Symptoms (ICIQ-MLUTS) Long Form and the Cleveland Clinic Florida Fecal Incontinence Score (CCFIS) together to assess pelvic floor function. The ICIQ-MLUTS Long Form consists of 23 symptom items, which include additional items compared to the short form such as bladder area pain, strain at the beginning of urination, urination posture, history of weak urinary stream, urinary stream force (picture question), urinary pain, terminal dribble, pad usage, urge to urinate again after voiding, and urinary piggybacking. According to the guidelines provided by the developers of the scale, items 11 and 13 are scored from 0 to 1, item 14 is scored from 1 to 4, items 21 and 23 are scored from 0 to 3, and the remaining items are scored from 0 to 4. The total score on the scale ranges from 1 to 84, with higher scores indicating more severe symptoms. Each symptom item also includes a sub-question regarding the level of bother caused by the symptom, which is scored from 0 to 10. This section is not included in the total score of the scale;  The CCFIS, also known as the Wexner score, was developed by Jorge in 1993 [26]. The CCFIS is widely used internationally as an assessment tool for evaluating the severity of fecal incontinence in patients [27]. It consists of five events, including solid stool incontinence, liquid stool incontinence, gas incontinence, pad usage, and lifestyle changes. Patients rate the frequency of each event’s occurrence based on their own experiences. The scoring ranges from 0 to 4, with 0 indicating "never occurred," 1 indicating "frequency<1 time/month," 2 indicating "frequency 1 time/month to <1 time/week," 3 indicating "frequency 1 time/week to <1 time/day," and 4 indicating "frequency ≥1 time/day." The total score ranges from 0 to 20, with 0 representing normal and 20 representing complete fecal incontinence. Higher scores indicate a greater severity of fecal incontinence. This study included participants who scored 9 or lower [28], and the frequency level for each item was limited to "sometimes." The test-retest reliability of this tool is 0.75. The tool is simple, objective, accurate, and easy to complete. Hui et al applied this tool in their study on intestinal symptoms in patients with ulcerative colitis [29]; |
| Communicative competence | The subjects can communicate normally; |
| Voluntarily participate | The subjects voluntarily participate in this study. |
| **Exclusion criteria** | |
| History of any type of urinary incontinence, fecal incontinence, urinary retention, chronic constipation, prolapse of important pelvic organs, and other diseases; | |
| History of abdominal or pelvic surgery in the past year, such as receiving physical therapy or surgery for urinary incontinence, or pelvic organ prolapse; | |
| Using medication that affects urinary incontinence or skeletal muscles in the past year; | |
| Received hormone replacement therapy in the past 6 months; | |
| Any bowel movements, mucous leakage, active urethral or vaginal infections in the past 3 months; | |
| Body mass index (BMI)＞35; | |
| Suffering from mental illness; | |
| Contraindications for MRI and CT examinations, such as intrauterine device insertion, pacemaker placement, and claustrophobia; | |
| CT findings of incomplete pelvic structure, fractures, osteoporosis, tumors, tuberculosis, rheumatism, pelvic masses, pelvic floor deformities, etc; | |
| Neurological and spinal cord diseases or injuries; | |
| With urethral obstruction, stenosis, or stones; | |
| Having a history of chronic cough, neuromuscular disease, and long-term weight-bearing work; | |
| Any other comorbidities or risk factors that interfere with the study. | |

## **References**

1. Li MM, Dai YJ. Progress in the Application of the Barthel Index Scoring Scale in Rehabilitation Nursing. Nursing Journal of Continuing Education. 2018, 33(6):508-510.[<https://kns.cnki.net/kcms2/article/abstract?v=s5eXW7nWjw0p31YaTqL7HyJvURlXdEGYkoXThBe8wPmD_GQUGlNgmAa0U3XkQrcK0DOa3lZZmh6XQM27FCgaLd8CzIAdx3B7aMOvs6fkbpbcAvArWhrdITesVK-h3D0Ql4hkjvX7uyR0BlJSXDsIxA==&uniplatform=NZKPT&language=CHS>] [doi: 10.16821/j.cnki.hsjx.2018.06.010]
2. Yeung PY, Wong LLL, Chan CC, et al. Montreal Cognitive Assessment-single cutoff achieves screening purpose. Neuropsychiatr Dis Treat. 2020,16:2681-2687. [https://www.dovepress.com/montreal-cognitive-assessment-mdash-single-cutoff-achieves-screening-p-peer-reviewed-fulltext-article-NDT] [doi: 10.2147/NDT.S269243] [Medline: 33192067]
3. Barber MD, Walters MD, Bump RC. Short Forms of Two Condition Specific Quality of Life Questionnaires for Women with Pelvic Floor Disorders(PFDI-20 and PFIQ-7). American Journal of Obstetrics&Gynecology. 2005,193(1):103-113. [https://www.ajog.org/article/S0002-9378(04)02108-8/abstract] [doi: 10.1016/j.ajog.2004.12.025] [Medline: 16021067]
4. Jorge JM, Wexner SD. Etiology and management of fecal incontinence. Dis Colon Rectum. 1993, 36(1):77-97. [https://journals.lww.com/dcrjournal/abstract/1993/36010/etiology_and_management_of_fecal_incontinence.16.aspx] [doi: 10.1007/BF02050307] [Medline: 8416784]
5. D'Amico F, Wexner SD, Vaizey CJ, et al. Tools for fecal incontinence assessment: lessons for inflammatory bowel disease trials based on a systematic review. United European Gastroenterol J. 2020,8(8):886-922. [https://onlinelibrary.wiley.com/doi/10.1177/2050640620943699] [doi:10.1177/2050640620943699] [Medline: 32677555]
6. Rothbarth J, Bemelman WA, Meijerink Wl, et al. What is the impact of fecal incontinence on quality of life?. Dis Colon Rectum. 2001:44:67-71. [https://link.springer.com/article/10.1007/BF02234823] [doi: 10.1007/BF02234823]
7. Hui H, Xu DY, Zhao HJ, et al. Investigation and analysis of functional intestinal symptoms in patients with remission ulcerative colitis. Chin J Dig Med Imageology Electron Ed. 2022, 12(1):16-19. [https://kns.cnki.net/kcms2/article/abstract?v=s5eXW7nWjw12s64RjDtBj04i5hfj7C0GEHwVPVIlc5qWNh6kom8hS8V7kRChiCdrONo94qTI40vU723ET4ryAs4Z3oFkWt4VkCauilnHibhaPHqiAVWnHA2sVh9zVaWPNgJucdyYiCO6TJouP-H4wA==&uniplatform=NZKPT&language=CHS]
