# Supplementary material for: Finite Element Analysis of Pelvic Floor Biomechanical Models to Elucidate the Mechanism for Improving Urination and Defecation Dysfunction in Older Adults: Protocol for a Model Development and Validation Study
Source: JMIR Res Protoc. 2024 May 31;13:e56333. doi: 10.2196/56333 (PMC11179018; doi:10.2196/56333)
Supplement: Multimedia Appendix 2 [file resprot_v13i1e56333_app2.docx]

**Table 1 Scanning methods of static MRI**

| **Steps** | **Methods** |
| --- | --- |
| Preparation | The night before the examination, the subject will squeeze a piece of glycerin into the anus to promote defecation. Check the light diet of the day and empty the stool and bladder before the examination. |
| Position | The subject is in a supine position, with their body in the middle of the bed and their legs extended and together. Their legs will be extended and kept together, while the body matrix coils are placed on the lower abdomen to minimize the artifacts caused by respiratory movements. |
| Time | Each subject's scan takes approximately 30-40 minutes. |
| Sequence method | 3D-PelvicVIEW-T2 scanning was used. |
| Scanning range | From the highest point of the iliac crest (or sacral promontory) to 1cm below the perineum, scan the entire pelvic floor, including pelvic floor muscles. |
| Layer thickness | A 3.0T magnetic resonance scanner produced by Philips in the Netherlands was used for 3D-PelvicVIEW-T2 scanning, with TR 1250 ms, TE 151 ms, and a field of view of 400 cm. In previous studies, the scanning thickness range of static MRI was 0.8 mm to 2.0 mm, including 60 to 192 layers，in this study, continuous volume scans with a thickness of 1.0 mm will be performed without a septum. |
| After scanning | Two doctors manually identify and outline the boundaries of the pelvis, muscles, ligaments, etc. in each layer of the image, and reach a consensus together. |
| Data saving | Export and save in DICOM format. |

**Table 2 Scanning methods of dynamic MRI**

| **Steps** | **Methods** |
| --- | --- |
| Pre experiment training | The literature indicates that dynamic MRI training can improve the success rate of scanning. Therefore, researchers will train participants to practice Kegel movements (anal lifting) and Valsalva movements to ensure good cooperation. |
| Starting position | The subject is in a supine position, with their body located in the middle of the bed surface and their legs extended and together. Their legs will be extended and kept together, while the body matrix coils are placed on the lower abdomen to minimize the artifacts caused by respiratory movements. |
| Estimated total time | Each participant is expected to complete the entire process in approximately 10 minutes. |
| Sequence method | According to literature review, fast imaging with steady state acquisition (FIESTA), single shot fast spin echo (SSFSE), and true fast imaging with steady state preparation (True FISP) were used, Half Fourier acquisition single shot turbo spin echo (HASTE) and other T2 weighted fast scanning sequences with extremely fast scanning speed are collected using half Fourier acquisition single shot turbo spin echo (HASTE). |
| Scanning range | up to the highest point of the iliac crest (or sacral promontory), down to 1cm of the perineum, complete pelvic floor scan, including pelvic floor muscles. |
| Layer thickness | According to the MRI technology and reporting standards for female pelvic floor functional disorders, it is found that single or simultaneous multiple layers (usually three consecutive layers) should be scanned in the midsagittal plane, with a steady-state sequence movie mode, a layer thickness of 8 mm, and a frame rate of 9-10 frames/s. |
| Actions that subjects should complete during dynamic MRI scanning | Pelvic floor dynamic MRI can quickly capture dozens of images in just over ten seconds, thereby achieving real-time dynamic observation of changes in the position of pelvic floor muscles and organs. After the static MRI scan is completed, instruct the patient to perform Kegel and Valsalva movements, and perform dynamic MRI scans while increasing abdominal pressure.  Based on relevant literature, the sequence of movements for the subjects is (1) resting phase (currently, patients are required to maintain 10 seconds, and it is recommended in the literature that a single dynamic cine scan time should not exceed 20 seconds for patients to hold their breath and cooperate); (2) Lifting the anus (Kegel action: lifting the anus, currently requiring patients to maintain it for 10 seconds); (3) Relaxation phase (currently, patients are required to maintain it for 10 seconds); (4) Valsalva maneuver (increasing abdominal pressure while holding breath and forcefully emptying the bladder and rectum, currently requiring patients to maintain it for 10 seconds) is used as a cycle, repeated 2-3 times per cycle. |
| Data saving | Export and save in DICOM format (medical digital imaging and communication). |

**Table 3 Scanning methods of CT**

| **Steps** | **Methods** |
| --- | --- |
| Preparation | None. |
| Position | The research subject is placed in a supine axial position. Try to be in the same posture as static MRI to facilitate the establishment of finite element models with MRI data in the later stage. |
| Time | Each subject takes approximately 5 minutes to scan. |
| Scanning range | The range is not less than the range of magnetic resonance imaging, from the highest point of the iliac crest to the knee joint. |
| Scanning process | The 64 row spiral scanning dual source CT machine produced by Siemens in Germany is used for thin layer scanning, with both layer thickness and scanning thickness of 0.5mm, 512 × 512 matrix. |
| Personnel | One skilled doctor is required to complete pelvic floor CT scanning examination; After the scan is completed, one doctor evaluates the quality of the collected imaging data and needs to ensure better quality. |
| Data saving | Export and save in DICOM format. |
