# Supplementary material for: Finite Element Analysis of Pelvic Floor Biomechanical Models to Elucidate the Mechanism for Improving Urination and Defecation Dysfunction in Older Adults: Protocol for a Model Development and Validation Study
Source: JMIR Res Protoc. 2024 May 31;13:e56333. doi: 10.2196/56333 (PMC11179018; doi:10.2196/56333)
Supplement: Multimedia Appendix 3 [file resprot_v13i1e56333_app3.docx]

**Table 1 Muscles corresponding to five rehabilitation trainings for four types of urinary and defecation dysfunction**

| **Types of urinary and defecation dysfunction** | **Rehabilitation trainings** | **Muscles** |
| --- | --- | --- |
| Urinary incontinence | Pelvic floor muscle training | 1. Pelvic floor muscle training: Pelvic floor muscle group; Urethral sphincter and levator ani muscle  2. Suspension exercise training: pelvic floor muscles+urethral sphincter and abdominal muscles and hip muscles+back muscles  3. Hip muscle exercise: pelvic floor muscle group and hip muscles |
|  | Electrical stimulation | Pelvic floor muscle group |
|  | Magnetic stimulation | Pelvic floor muscle group |
|  | Biofeedback | Pelvic floor muscle group; Levator ani muscle |
|  | Vibrational stimulation | Pelvic floor muscle group |
| Uroschesis | Pelvic floor muscle training | 1. Pelvic floor muscle training: Pelvic floor muscle group; Urethral sphincter and levator ani muscle  2. Hip muscle exercise: pelvic floor muscle group and hip muscles |
|  | Electrical stimulation | Pelvic floor muscle group and urethral sphincter |
|  | Magnetic stimulation | Pelvic floor muscle group |
|  | Biofeedback | Pelvic floor muscle group; Levator ani muscle |
|  | Vibrational stimulation | None |
| Fecal incontinence | Pelvic floor muscle training | Pelvic floor muscle group; Pelvic floor muscle group and external anal sphincter |
|  | Electrical stimulation | Sacral nerve anterior root electrical stimulation: pelvic floor muscle group; Pelvic floor muscle group and external anal sphincter |
|  | Magnetic stimulation | Pelvic floor muscle group; Pelvic floor muscle group and abdominal muscles+back muscles |
|  | Biofeedback | Pelvic floor muscle group; Pelvic floor muscle group and external anal sphincter |
|  | Vibrational stimulation | none |
| Constipation | Pelvic floor muscle training | Pelvic floor muscle group; Levator ani muscle and external anal sphincter |
|  | Electrical stimulation | Pelvic floor muscle group |
|  | Magnetic stimulation | Pelvic floor muscle group; Levator ani muscle and external anal sphincter |
|  | Biofeedback | Pelvic floor muscle group; Levator ani muscle and external anal sphincter |
|  | Vibrational stimulation | Pelvic floor muscle group |

Note: The pelvic floor muscle group includes the bulbocavernosus muscle, ischiocavernosus muscle, superficial transverse perineal muscle, external anal sphincter, deep transverse perineal muscle, urethral sphincter, levator ani, and coccyx muscle. The levator ani muscle includes the pubococcygeus muscle, iliococcygeus muscle, and puborectalis muscle. In this study, the abdominal muscle specifically refers to the rectus abdominis muscle, while the hip muscle specifically refers to the iliopsoas muscle, quadriceps femoris muscle, gluteus maximus muscle, hamstring muscle, gluteus medius muscle, and adductor longus muscle. In this study, the back muscle specifically refers to the erector spinae muscle.
